# Combined Pulsed Vacuum Osmotic Dehydration and Convective Air-Drying Process of Jambolan Fruits

**DOI:** 10.3390/foods12091785

**Published:** 2023-04-25

**Authors:** Adriano Lucena de Araújo, Rosinelson da Silva Pena

**Affiliations:** 1 Graduated Program in Food Science and Technology (PPGCTA), Institute of Technology (ITEC), Federal University of Pará (UFPA), Belém 66075-110, PA, Brazil; 2 Faculty of Food Engineering (FEA), Institute of Technology (ITEC), Federal University of Pará (UFPA), Belém 66075-110, PA, Brazil

**Keywords:** osmosis, pretreatment, preservation, bioactive compounds

## Abstract

Jambolan (*Syzygium cumini*) is a native fruit from Asia that has adapted well to the tropical climate of the Amazonian region. However, due to its limited annual availability and high perishability, the jambolan fruit is still underexploited. Thus, this study aimed to preserve the jambolan through a combined process of pulsed vacuum osmotic dehydration (PVOD) and convective air-drying and to monitor the total phenolic contents (TPCs) and total monomeric anthocyanins (TMAs) during these processes. To this end, jambolan fruits were pretreated with increasing PVOD times. After monitoring of moisture loss, solid gain, weight reduction, water activity, TPC, and TMA, pretreated (PT) and non-pretreated (NPT) fruits underwent convective air-drying (50–70 °C). The PVOD reduced half of the water present in the fruits; nonetheless, PVOD decreased the TPC and TMA over time. The increase in air-drying temperature shortened the drying time for both NPT and PT jambolan, and PVOD reduced even further the drying time of the fruits. Moreover, the fruits pretreated and dried at 60 °C showed promising results, potentially being a good alternative to extend the fruit’s shelf life and make it available throughout the year.

## 1. Introduction

Phenolic compounds are secondary metabolites with protective roles in higher plants. They are mainly produced using the shikimic acid pathway and can be divided into many subgroups, most likely phenolic acids, tannins, and flavonoids [1]. Among flavonoids, anthocyanins represent one of the most abundant constituents, corresponding to the red, blue, and purple colors of fruits and vegetables. These pigments can degrade during thermal processing, which can influence the color quality and affect the nutritional properties of the product [2].

The ingestion of foods rich in anthocyanins has been associated with some health benefits, which can induce positive effects in the organism, because of the bioactivity of these substances [3]. In this scenario, jambolan (*Syzygium cumini* (L.)) fruit appears as a potential source of phenolic compounds, being also rich in anthocyanin pigments [4]. Its potential therapeutic benefits can be explained by its antioxidant and antimicrobial properties [1].

Moreover, jambolan is an ellipsoid berry (3–5 cm length) that presents a whitish/pinkish pulp, reaching full ripening when its peel manifests a purple to black color [5]. Native from India, it was first introduced in Brazil by Portuguese settlers and commonly spread in nature. The fruit does not have a high commercial value in Brazil, and most of the production is lost [6]. Moreover, jambolan is a seasonal fruit that is only available in the months of December to February, and its shelf life is no longer than 2 days at room temperature [7].

Throughout post-harvesting, fresh fruits become more susceptible to several changes that lead them to senescence and deterioration by biochemical reactions and microbial growth. These two variables represent the most common limiting factors that influence the food’s shelf life. Thus, preservation techniques are needed to achieve stability and maintain the moisture content under levels required for fruit conservation [8].

Dehydration is a common preservation technique for reducing post-harvest losses, and this process can extend the shelf life and availability of fruits during all seasons [9]. Hence, to improve the quality of preserved food products, researchers have aimed for alternative paths to process foods, and one of these methods is osmotic dehydration (OD) [10]. OD is usually applied as a pretreatment before drying operations, since it involves the immersion of foods in hypertonic solutions for partial water removal, due to the pressure difference between the food and the hypertonic solution [11].

This preservation technique is simple and facilitates the processing of fruits, concerning the retention of initial characteristics, such as color, aroma, texture, and nutritional composition [12]. However, OD will not provide a safe moisture content to be considered as a shelf-stable product, and therefore, the osmotically dehydrated product must be further dried through some complementary process, such as air, vacuum, or freeze-drying [13].

Mass transfer during OD depends on some factors, including the product’s geometry, temperature, agitation, type, and concentration of the osmotic agent, among others [14,15,16,17]. Recently, calcium salts have been employed to reduce damages caused by the cell wall structure and improve the mass transfer of the osmo-dehydrated products [18,19]. In addition, the increment of pulsed vacuum osmotic dehydration (PVOD), at the beginning of the OD process, has been used as a resource to accelerate the mass transfer in plant tissues [20,21,22,23,24].

There have been PVOD kinetics studies related to jambolan, using ultrasonic vacuum pretreatment [25], as well as other research emphasizing the selection of variables with significant effects over the fruit’s OD [26]. However, as far as our knowledge is concerned, no studies have contemplated the use of OD pretreatment combined with air-drying conditions for this fruit. Thus, the main goal of this research was to assess the preservation of the jambolan fruit through PVOD and convective drying and, at the same time, to monitor the total phenolic contents (TPCs) and total monomeric anthocyanins (TMAs) during osmotic pretreatment and the air-drying process.

## 2. Materials and Methods

### 2.1. Plant Material

Jambolan (*S. cumini*) fruits were collected from November to December 2021 from local trees at the Federal University of Pará (UFPA) (latitude 1°28′ S, longitude 48°29’ W) in the city of Belém (Brazil). The harvested fruits were transported in polystyrene isothermal boxes to the Laboratory of Fruits, at the same university. After that, mature and integral fruits were chosen as a selection criterion for further analysis. Then, these fruits were rinsed in running water and sanitized by immersion in hypochlorite solution at 20 mg/L of active chlorine for 15 min, followed by a second rinsing. Furthermore, the sanitized fruits were packaged in low-density polyethylene packages and placed in polypropylene pots coated with aluminum foil. The fruits were stored at −18 °C, until the next procedure.

### 2.2. Osmotic Pretreatment

The following set was chosen for the osmotic pretreatment: 60% sucrose, 4% calcium lactate, and 10 kPa absolute pressure (90% vacuum pulse). These conditions presented the greatest moisture losses in jambolan, according to the previous study from Araújo and Pena [26]. Additionally, to avoid significant depletion of bioactive compounds during PVOD (at elevated temperatures) [27], the set was fixed at 25 °C. The osmotic solutions were prepared by dissolving sucrose and calcium lactate in distilled water, at ambient temperature (≈25 °C).

Initially, jambolan fruits were thawed (at room temperature) and weighed on an analytical balance (M214AIH, BEL, Brazil). Furthermore, each fruit was put in a beaker with the osmotic solution, using the fruit/solution ratio of 1:10 (*m*/*v*). After that, the beakers (quadruplicates) were positioned inside a glass desiccator at room temperature (≈25 °C). Then, the system was sealed and submitted to the specific pressure (vacuum pulse) for five minutes. A vacuum pump (131, Prismatec, Brazil) was used to guarantee pressure control. At the end of the pressure time, the system’s vacuum was interrupted, and the set was maintained under atmospheric pressure until the total process time was finished. To keep a constant temperature, the beakers (containing the fruits) were coated with aluminum foil and placed in a biochemical oxygen demand (BOD) incubator (Q315M16, Quimis, Brazil), at 25 °C (±1 °C).

PVOD samples were taken from osmotic solution after different treatment times: 1, 4, 8, 16, 24, 48, and 72 h; rinsed with 20 mL of distilled water; and dried for 10 s in an absorbent paper to remove sucrose/calcium lactate and water in excess from the fruit surface. Then, the PVOD samples were weighed again in the analytical balance.

### 2.3. Convective Air-Drying Kinetics

Non-pretreated (NPT) and osmotically pretreated (PT) samples were submitted to air-drying in a convective tray dryer (Q-316M5, Quimis, Brazil) at 50, 60, and 70 °C. Samples were weighed in a semi-analytical balance (S203H, BEL, Italy) (± 0.001 g) for monitoring purposes. During the first 60 min of drying, samples were weighed every 20 min, and after 60 min of drying, samples were weighed every 60 min until the sample’s mass variation was below 0.1% (equilibrium condition). This procedure was performed in three replicates. The drying curves were produced on the basis of the correlation between moisture ratio (MR) (Equation (1)) and the drying time (t). The moisture content was assessed before and after drying.

(1) $$\mathrm{MR}=\frac{{m\;-\;m}_{e}}{m_{i}\;{-\;m}_{e}}$$

where MR = moisture ratio (dimensionless), and m, m_i_, and m_e_ are the moisture contents at a given t time, initial, and equilibrium (g/g dry basis—db), respectively.

### 2.4. Monitoring Analyses

Monitoring analyses (moisture content, water activity, total monomeric anthocyanins, and total phenolic contents) were assessed in the non-pretreated sample (control) and in the dried samples with and without osmotic pretreatment. During each point of the PVOD kinetic, the mentioned monitoring analyses were performed, including moisture loss, solid gain, and weight reduction.

#### 2.4.1. Moisture Loss, Solid Gain, and Weight Reduction

Moisture loss (ML), solid gain (SG), and weight reduction (WR) were calculated according to Sridevi and Genitha [28] by Equations (2)–(4).

(2) $$\mathrm{ML}(\%)=\frac{{(W}_{0\;}{-\;W}_{t})}{M_{0}}\times 100$$

(3) $$\mathrm{SG}(\%)=\frac{{(S}_{t}{\;-\;S}_{0})}{M_{0}}\times 100$$

(4) $$\mathrm{WR}(\%)=\frac{{(M}_{0\;}{-\;M}_{t})}{M_{0}}\times 100$$

where W_0_ = initial water mass of the sample (g); W_t_ = water mass of the sample (g) after dehydration; S_0_ = initial dry mass of the sample (g); S_t_ = dry mass of the sample (g) after dehydration; M_0_ = initial mass of the sample (g); M_t_ = mass of the sample (g) after dehydration.

#### 2.4.2. Water Activity and Moisture Content

The water activity (a_w_) was determined by direct reading in the water activity meter (Aqua LAB 4TEV, Decagon Devices, USA), at 25 °C. Moisture content (MC) was determined by drying the sample at 105 °C, according to the Association of Official Analytical Chemist [29].

#### 2.4.3. Total Monomeric Anthocyanins and Total Phenolic Contents

An extract was prepared for both essays of total monomeric anthocyanins (TMA) and total phenolic contents (TPC). The extraction followed the procedure from Brito et al. [30], with modifications. The sample (≈0.3 g) was exhaustively macerated with acidified ethanolic solvent (EtOH_95%_/HCl_1,5M_, 85:15, *v*/*v*) using a mortar and a pestle. Then, the macerated sample was put inside a conical centrifuge tube Falcon (15 mL), and centrifuged at 1980× *g* for 5 min in a centrifuge (K14-0815C, Kasvi, São José dos Pinhais, Brazil). After that, the supernatant was removed from the tube with a graduated pipette, transferred to a volumetric flask (10 mL), and completed with the extraction solvent. The extract concentration was equivalent to 0.03 g/mL, which corresponds to 30 g/L.

TPC was determined through the method described by Singleton and Rossi [31], on the basis of the reaction with the Folin–Ciocalteau reagent. Quantification was made by reading in a UV-VIS spectrophotometer (Evo 60, Thermo-Fisher Scientific, Waltham, MA, USA), at 760 nm. Concentrations between 5 to 100 mg gallic acid/L were used for the construction of the analytical curve. TPC was expressed as mg of gallic acid equivalent (GAE)/100 g fruit (db).

TMA was determined according to Giusti and Wrolstad [32], which is based on the quantification of monomeric anthocyanins by the differential pH method. Briefly, aliquots of the extract were added to each buffer: 0.025 M potassium chloride (pH 1.0) and 0.4 M sodium acetate (pH 4.5). The absorbance of the mixture was measured at 514 nm and 700 nm, using the UV-VIS spectrophotometer. Absorbance (A) was calculated as A = [(A_514nm_ − A_700nm_) at pH 1.0] − [(A_514nm_ − A_700nm_) at pH 4.5]. The TMA was calculated as delphinidin-3-glucoside equivalent (Equation (5)). The results were converted to mg delphinidin-3-glucoside/g by dividing the TMA value (mg/L) by the extract concentration (30 g/L). Then, TMA results were expressed as mg delphinidin-3-glucoside equivalent/100 g fruit (db).

(5) $$\mathrm{MA}(\mathrm{mg}/L)=\frac{{A\;\times \;\mathrm{MW}\;\times \;\mathrm{DF}\;\times \;10}^{3}}{\varepsilon \;\times \;\lambda }$$

where MW = molecular weight (465 g/mol of delphinidin-3-glucoside); DF = dilution factor; Ԑ = molar attenuation coefficient (29,000 L/mol.cm); λ = length of the cuvette (1 cm); 10^3^ = conversion factor from g to mg.

### 2.5. Pulsed Vacuum Osmotic Dehydration Modeling

#### 2.5.1. Azuara’s Model

A two-parameter model was used to predict the PVOD kinetic of jambolan. The values of ML and SG, at equilibrium, and the k parameter were calculated by linear regression from the relationship t/GX versus t, according to the linearized form of the model proposed by Azuara et al. [33] (Equation (6)).

(6) $$\frac{t}{\mathrm{GX}}=\frac{1}{{k\times \mathrm{GX}}^{\infty }}+\frac{1}{{\mathrm{GX}}^{\infty }}\;t$$

where GX = moisture loss (ML) or solid gain (SG) (%) in a time t; GX^∞^ = moisture loss (ML^∞^) or solid gain (SG^∞^) (%) at equilibrium; k = constant (h^−1^); and t = time of the process (h).

#### 2.5.2. Peleg’s Model

Peleg’s model was set to fit the experimental data of PVOD jambolan. It is an empirical model with two parameters, initially used to describe curves that approach equilibrium [34], according to Equation (7). For a condition of t → ꝏ, the values of moisture loss (ML^ꝏ^) and solid gain (SG^ꝏ^), at equilibrium, were estimated from the 1/k_2_ ratio of each respective process.

(7) $${\mathrm{WX}=\mathrm{WX}}_{0}\pm \frac{t}{k_{1}{+k}_{2}}\;$$

where WX = moisture loss (ML) or solid gain (SG) (%) in a time t; WX_0_ = initial ML or SG (%); k_1_, k_2_ = Peleg’s constants; and t = time of the process (h).

The fits of the models to the experimental data were confirmed by the coefficient of determination (R^2^) and by the root mean square error (RMSE) (Equation (8)).

(8) RMSE=[1n∑i-1n(mexp,i - mpre,i)2]12

where m_exp,i_ = experimental ML or SG value; m_pre,i_ = ML or SG value predicted by the model; and n = number of observations.

### 2.6. Calculation of Effective Moisture Diffusivity

The effective moisture diffusivity (D_eff_) from the air-drying process for NPT and PT jambolan was estimated according to the analytical solution of Fick’s second law, in the unsteady state, for nearly spherical materials (Equation (9)) [35]. The calculation was conditioned by assuming that the moisture transfer is predominantly one-directional; the initial moisture content is equally diffused in the product, shrinkage is negligible, and the diffusion coefficient is assumed constant and homogeneous during drying.

(9) $$\mathrm{MR}=\frac{6}{\pi^{2}}\overset{\infty }{\underset{n=1}{\sum }}\frac{1}{n^{2}}\exp\left({-n}^{2}\pi^{2}\frac{D_{\mathrm{eff}}t}{r^{2}}\right)$$

where D_eff_ = effective moisture diffusivity (m^2^/s); r = radius of a sphere (m); n = positive integer; and t = time (s).

Simplifying Equation (9) and taking only the first term of the series into consideration, it could be rewritten in a logarithm form as Equation (10), which is an acceptable approximation in longer times (setting n = 1).

(10) $$\mathrm{lnMR}=\ln\left(\frac{6}{\pi^{2}}\right)-\left(\frac{\pi^{2}D_{\mathrm{eff}}}{r^{2}}\right)t$$

The plot of experimental drying data of ln (MR) versus time (t) produced a straight line with a slope (S), and the D_eff_ was calculated in Equation (11) by linear regression [20].

(11) $$D_{\mathrm{eff}\;}=S{\left(\frac{d_{e}}{2\pi }\right)}^{2}$$

where S = slope of ln (MR) versus drying time (t), and d_e_ = geometric mean diameter (m) of the fruit.

The shapes of NPT jambolan and PT jambolan were assumed as ellipsoidal, and the geometric mean diameters (d_e_) were obtained from the three measured diameters, as presented in Equation (12) [36].

(12) $$d_{e}=\sqrt[{3}]{d_{x}d_{y}d_{z}}$$

The effect of air-drying temperature on the D_eff_ of NPT and PT jambolan was estimated by an Arrhenius-like equation (Equation (13)) [37].

(13) $$D_{\mathrm{eff}}{=D}_{0}\exp\left(-\frac{E_{a}}{\mathrm{RT}}\right)$$

where E_a_ = activation energy (kJ/mol); D_0_ = pre-exponential factor of the Arrhenius equation (m^2^/s); R = universal gas constant (8.31 kJ/mol), and T = absolute air temperature (K).

### 2.7. Drying Kinetics Modeling

Table 1 presents the drying classic models [38] used to predict the air-drying kinetics for NPT and PT jambolan.

Mathematical modeling of the drying curves was performed by non-linear regression using Statistica software 7.0. The Levenberg–Maquardt algorithm was employed with a convergence criterion of 10^−6^. The statistics R^2^, RMSE (Equation (8)), reduced chi-square (χ^2^) (Equation (23)), and residues dispersion (RD) were used to select the best drying fits.

(23) $$\chi^{2}=\frac{\sum_{i=1}^{N}{{(\mathrm{MR}}_{\exp,i\;}{-\;\mathrm{MR}}_{\mathrm{pre},i})}^{2}}{N\;-\;n}$$

where MR_exp,i_ and MR_pre,i_ are moisture ratios determined from the experimental data and predicted data by the fitted models, respectively; N = number of experimental measurements; and n = number of model parameters.

## 3. Results and Discussion

### 3.1. Pulsed Vacuum Osmotic Dehydration Kinetic

#### 3.1.1. Changes in Moisture Loss, Solid Gain, Weight Reduction, and Water Activity

**Figure 1:**
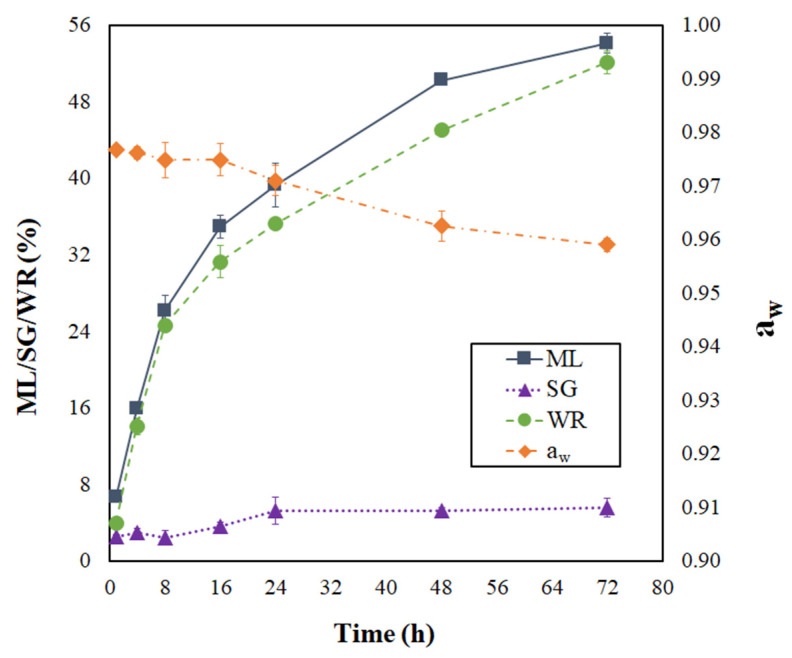
Progressing of moisture loss, solid gain, weight reduction, and water activity during the PVOD kinetic of jambolan fruits.

The progress of moisture loss (ML), solids gain (SG), weight reduction (WR), and water activity (a_w_) during the PVOD kinetic of jambolan can be visualized in Figure 1. As can be seen, ML and WR presented a sharp increase in the first 16 h of the process, which represented 64.5% and 60% of the total ML and WR accounted for jambolan, respectively, during its PVOD kinetic. After that period, ML and WR evolution became less intense, until no big changes were observed. The osmotic pressure is the driving force responsible for water diffusion from the food to the solution, which is higher at the beginning of the process and promotes higher ML rates due to the difference between concentrations of the dilute solute (from the fruit) and its surrounding hypertonic solution [25,39,40]. In contrast, SG rates were not prominent at the initial stages of osmotic treatment (first 8 h). However, SG slightly increased in the material after 8 h. Thus, the pressure and concentration gradients gradually decreased as time progressed, until the equilibrium condition was achieved.

ML features a key role in OD processes because it generates osmotic shock in microbial cells, which might affect their osmoregulation. Microorganisms keep the pressure turgor by retaining slightly lower moisture content (MC) inside their cells, in comparison to the outside environment. When OD is applied by either removing water or adding solutes or hydrophilic colloids, the environment’s free water is reduced, which causes the free water of microorganism cells (hypotonic) to flow from the inside to the exterior of the cells (hypertonic environment) and may result in plasmolysis of microorganism cells [41].

As expected in OD processes, ML was much higher than SG. After 72 h of process at 25 °C, ML and SG were around 54.10 ± 1.02% and 5.56 ± 0.96%, respectively. Sharma and Das [25] reached ML values around 35.2%, 47.1%, and 54.5%, under atmospheric pressure (AP), vacuum pretreatment (VP), and ultrasound vacuum pretreatment (USVP), respectively, during the OD of jambolan. For SG, the same authors observed the following values: 4.6% (AP), 7.6% (VP), and 9.3% (USVP). These ML and SG findings were reached after 5 h of the OD process, at 30 °C. On the other hand, the ML and SG values found by Araújo and Pena [26] varied from −1.06 to 21.35% and from −5.11 to 7.09%, respectively, in PVOD jambolan fruits, after 1.5 h of process, in treatments with different combinations of temperatures (20–50 °C), sucrose concentrations (30–60%), pressures (10–90 kPa), and calcium lactate concentrations (0–4%).

By increasing the PVOD time, a_w_ presented an important reduction (Figure 1). In the first 16 h of PVOD, the a_w_ values almost remained steady and, after that period, the a_w_ continually dropped until it reached nearly an equilibrium condition (72 h). Although the a_w_ reduction, from the numerical point of view, presented a slight decrease during the PVOD kinetic, a considerable change occurred in the fruit. Small a_w_ changes provoke considerable differences in the MC values at elevated a_w_ ranges, as shown by Araújo and Pena [42], during the moisture desorption process of jambolan pulp at 25 °C. These authors noticed that great amount of water was demanded to reduce the fruit’s initial a_w_.

In addition, a reduction in a_w_ decreases the lag phase of microorganisms and, thus, decreases their growth rate. However, some microorganisms present effective mechanisms to breakthrough plasmolysis, regain turgor by activating osmoregulation capacities, and maintain homeostasis [41]. Due to this fact, it is important to avoid microbial growth by lowering even further a_w_ with another complementary process (e.g., drying) in order to stabilize jambolan fruit under the required limits for food safety. Still, as shown in Figure 1, the a_w_ variations were proximate to those found in OD kinetics (30 °C) with sweet potato, using sucrose (51% concentration) as the osmotic agent [43].

**Figure 2:**
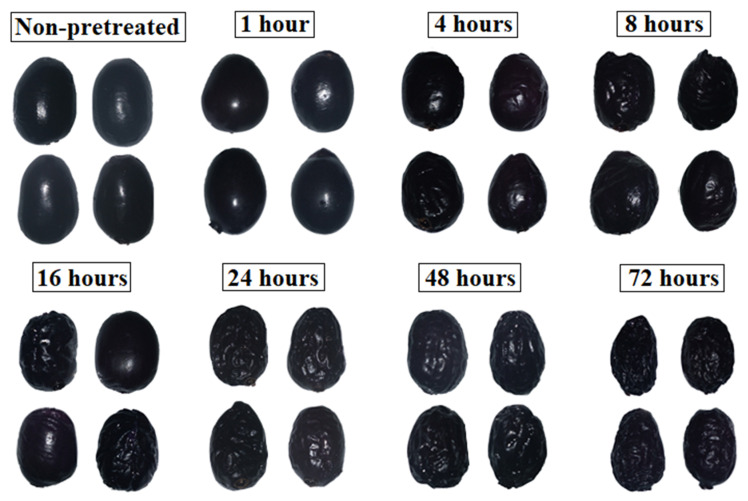
Overall appearance of jambolan fruits after different PVOD times.

Figure 2 shows the aspects of jambolan fruits at different PVOD times. During osmotic pretreatment, the shrinkage effect becomes more evident as time progresses. These modifications were observed in osmo-dehydrated cranberries as well and became more intense as the power of the microwave vacuum pretreatment increased from 100 to 800 W [44]. Yu et al. [45] noticed similar behavior after submitting blueberries to OD (40 °C), but the highest shrinkage effect was evidenced in samples pretreated with pulsed electric fields, with regards to those thermally pretreated.

The shrinkage occurs when a plant cell is placed in an environment with low water potential (e.g., sucrose solution). As a result, the vacuole and the rest of the protoplast retract and cause the detachment between the plasma membrane and the cell wall, which is known as plasmolysis. The mass transfer and shrinkage phenomena are diffused from the surface of the material to its center, as a function of the operation time [46].

#### 3.1.2. Total Monomeric Anthocyanins and Total Phenolic Contents

**Figure 3:**
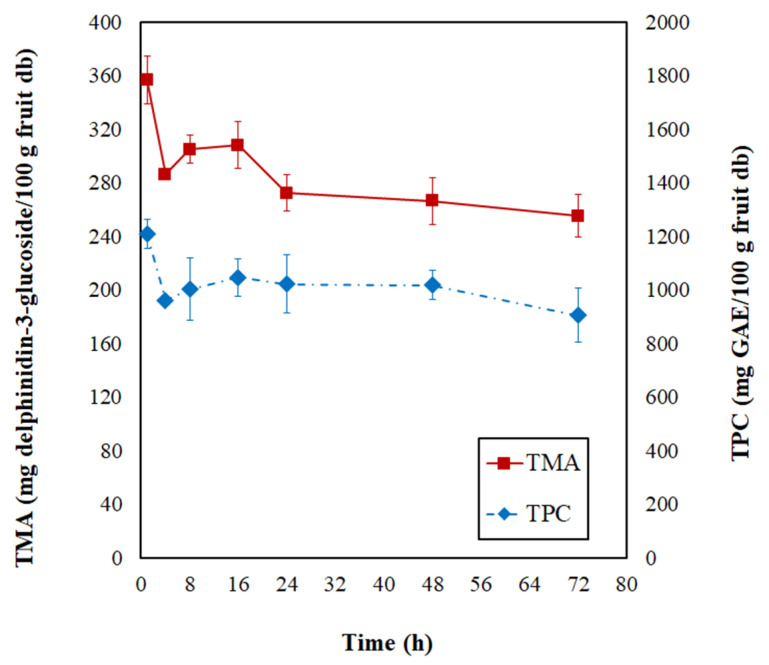
Changes of total monomeric anthocyanins (TMA) and total phenolic contents (TPC) during the PVOD kinetic of jambolan fruits.

In Figure 3, the total monomeric anthocyanin (TMA) and total phenolic content (TPC) modifications are evidenced during the PVOD kinetic of jambolan. During the first 4 h of the process, the TMA and TPC suffered a significant decrease, and, after this process time, the values remained almost stabilized until it reached the end of the operation (72 h). At the final PVOD time, TMA retained 53.43% and TPC retained 73.11%, concerning the initial contents.

Nowicka et al. [47] noticed a similar behavior throughout the OD kinetic of sour cherries at 40 °C. During the first 1 h, significant losses of TPC occurred, and the authors associated this with an intensive mass exchange during the first phase of OD, which was followed by a slight TPC increase in the second hour of OD, and further, it resulted in the TPC degradation over time by the loss of cell membrane selective transport of substances. Araya-Farias et al. [48] figured out that OD at 40 °C (during 6 h) caused an 88% reduction in TPC of seabuckthorn fruit. Sójka et al. [49] evidenced substantial decreases in the TMA contents of blackberries after 1 h of OD at 30 °C, in which retentions varied from 60.8 to 68.6%. These researchers realized that further OD time had a less dramatic effect on TMA, which also translated to the retention levels (50.5–55.9% and 46.8–53.6%, after 3 h and 5 h, respectively), at different osmotic solution concentrations (50, 57.5, and 65%).

During OD, the osmotic pressure difference results in three simultaneous flows: the water flow from the food matrix to the solution, the reverse flow of solutes from the concentrated solution to the product, and the removal of soluble solids from the product (e.g., vitamins, minerals, and other water-soluble nutrients) from the food into the solution [12]. Thus, chemical compounds present in fruits can be influenced by different variables of OD and the content of these compounds might change through biochemical or chemical transformations (e.g., enzymatic, hydrolytic, polymerization, biosynthesis) or even by their leaching into the osmotic solution. Therefore, further analysis of individual compounds can shed light on this question. Nonetheless, the ML phenomenon promotes the concentration of chemical compounds available in the raw material. On the contrary, SG increases the sample’s weight with an apparent decrease in chemical compounds [50]. Thus, these different pathways can affect the product’s final constituents. During OD at 40 °C of blueberries, Yu et al. [45] observed both the physical migration of bioactive compounds to the syrup medium and the degradation of these nutrients by biochemical reactions.

#### 3.1.3. Pulsed Vacuum Osmotic Dehydration Modeling

In the present study, the Azuara model (Table 2) better described the changes in ML and SG during the PVOD of jambolan. Although Peleg’s model presented lower RMSE results, the Azuara’s model presented higher R^2^ values, especially for SG (Table 2). Thus, it indicates that the Azuara’s model can be used with good accuracy to predict the ML and SG kinetic of jambolan fruits in the experimental domain. This model also displayed satisfactory fits (R^2^ > 0.96 and RMSE < 0.08) for ML and SG in osmotically dehydrated cherry tomatoes, at different salt concentrations (10–25%) and temperatures (30–40 °C) [51].

The k value of the Azuara’s model represents the process velocity when it reaches the equilibrium state; therefore, the higher the k value, the higher the water or solid diffusion by time unity. Thus, the k values showed that SG (k = 0.17 h^−1^) reached equilibrium faster than ML (k = 0.09 h^−1^). In addition, ML^∞^ and SG^∞^ parameters manifested ML and SG at equilibrium state, respectively. The values of these parameters showed that times beyond 72 h can contribute to higher water removal (ML^ꝏ^ = 61.7%) and little solid impregnation rates (SG^ꝏ^ = 5.95%), during PVOD of jambolan fruits.

### 3.2. Drying Kinetics

#### 3.2.1. Drying Kinetics of Pulsed Vacuum Osmotically Dehydrated Jambolan

**Figure 4:**
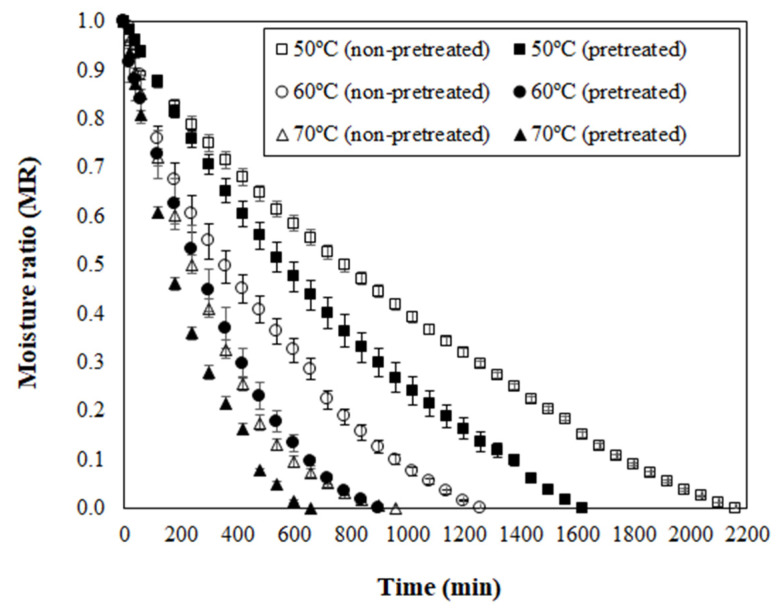
Moisture ratio (MR) versus drying time for non-pretreated and pretreated jambolan fruits submitted to drying at 50, 60, and 70 °C.

For the next discussions, the osmotically pretreated sample was defined on the basis of the highest ML value and the lowest a_w_ level, which matches with 72 h of PVOD (Figure 1). Curves of moisture ratio (MR) versus drying time for non-pretreated (NPT) and pretreated (PT) dried jambolan are shown in Figure 4, along with the temperature effect. The drying curves showed that the MR decreased with the drying time for all treatments. At the early stages of drying, the MR decreased relatively fast and, after reaching a certain time, it decreased slowly until it reached an equilibrium condition. For NPT jambolan, the times needed to reach the equilibrium moisture content was equivalent to 36 h at 50 °C, 21 h at 60 °C, and 16 h at 70 °C. In turn, for the PT jambolan fruits, the equilibrium times were equal to 27 h at 50 °C, 15 h at 60 °C, and 11 h at 70 °C.

The long drying periods noticed in the present research are awaited, since fruits are regularly difficult to dehydrate during hot air, due to their high-water content, which results in extensive drying times and subsequently leads to structural and color changes [52]. It is worth mentioning that the present study was set in whole jambolan fruits, and it is more challenging to conduct drying experiments in these conditions, considering that the fruit still contains its pericarp, whose natural feature is to protect the fruit from exterior influences. Therefore, it could difficult the water exits from the material, in comparison to sliced or cut fruits.

Furthermore, an increase in temperature shortened the drying time for both NPT and PT jambolan. This increase was expected since a higher air-drying temperature promotes higher moisture evaporation rates at the interface between the food and the surrounding air. The greater moisture evaporation leads the moisture to diffuse at elevated rates, from the internal regions of the material to the surface [53]. Potosí-Calvache et al. [54] inferred that dehydration at higher temperatures was more advantageous with regards to energy savings for squash.

Great amounts of ML during PVOD (Figure 1) led to significant decreases in the drying time of PT samples when compared to the NPT ones (Figure 4). The PT jambolan took about 25%, 29%, and 31% less time to be dried at 50 °C, 60 °C, and 70 °C, respectively. Thus, much less moisture content is demanded for its drying, which results in less required drying time to remove water from the material when compared to NPT jambolan. The effect of OD on the shortening of the drying time was already evidenced in the works of Sahin and Öztürk [20] and An et al. [55] during the drying of figs and cherry tomatoes, respectively. Fito et al. [56] stated that vacuum impregnation, during OD, could cause modifications in certain food’s structure and composition, which might increase the heat and mass transfer rates during air-drying operations.

#### 3.2.2. Effective Moisture Diffusivities and Activation Energy for Drying Process

The effective moisture diffusivities (D_eff_) for the air-drying process of NPT and PT jambolan fruits are presented in Table 3. These values lay within the range of food materials, which is from 10^−11^ to 10^−9^ m^2^/s [57]. Comparable results have been reported in the drying kinetics of fruits with spherical dimensions, such as Turkish berries (3.73 to 7.56 × 10^−10^ m^2^/s) [58], blueberries (2.58 to 4.58 × 10^−11^ m^2^/s) [59], and cranberries (0.92 to 2.37 × 10^−9^ m^2^/s) [44].

From the data, it is possible to infer that an increase in drying temperature had increased the D_eff_ during the drying of NPT and osmotically pretreated jambolan. According to Falade and Oyedele [60], this behavior is due to a higher driving force, which accelerates the transfer of water vapor from the inside to the surface of the product, promoting higher D_eff_ values. Hence, it indicates that higher temperatures can promote faster drying speed, which could be attributed to an increase in the heating energy [61]. In addition, the osmotic pretreatment resulted in higher D_eff_ values when compared to each respective temperature of the NPT samples. This same behavior was already evidenced in the drying of figs [20] and cherry tomatoes [55], both submitted to previous OD treatments.

In regard to the activation energy (E_a_) (Table 3), NPT samples presented higher values than the PT ones. In the literature, similar results have been observed for NPT (44.4 kJ/mol) and PT (32.02 kJ/mol) strawberries [62]. The E_a_ can be defined as the energy needed to displace one mol of water from the sample that is dried [59]. In this sense, the osmotic pretreatment has reduced the E_a_ demanded to dry the PT jambolan fruits. Still, some researchers have claimed that the lower the E_a_ in the drying process, the greater the D_eff_ of water in the product, which demands less thermal energy to enable the physical transformation of liquid water [63]. The E_a_ values observed lie within the general range for drying food products, which is comprehended between 12.7 and 110 kJ/mol [64].

#### 3.2.3. Drying Kinetics Modeling

Mathematical modeling is the process of creating a mathematical representation of a real-world scenario, which can be used for scaling up the processing capacity or even predicting its outcome. An equation with a sufficient approximation level could be employed to predict the drying time needed to obtain a desired moisture content [65]. The optimal criteria to analyze the quality of the models was based on the highest values for R^2^, lowest values for χ^2^, and RMSE, followed by a random distribution (RD) of residues. The data obtained for these statistics are displayed in Table 4 and describe the fits to the drying processes for NPT and PT jambolan at 50 °C, 60 °C, and 70 °C. Overall, the R^2^ values were higher than 0.97, the χ^2^ ranged from 1 × 10^−5^ to 2.5 × 10^−3^, and the RMSE values were between 4 × 10^−3^ and 5 × 10^−2^.

Although these model adjustments were within the previous optimal criteria defined previously, the RD must be verified as well. Through an analysis of scatterplots from residuals versus the predicted values, it can be observed if the residuals were randomly distributed around zero with no systematic patterns, at the significance level analyzed [66,67]. By these means, only Midilli’s model presented a random distribution of residues in all treatments studied, which indicates the goodness of fit, besides being able to predict the drying data. Still, this model has evidenced the most elevated R^2^ values (R^2^ = 1) and the lowest χ^2^ (<0.00004) and RMSE (<0.01) values, among all equations tested (Table 4). In the literature, Midilli’s model displayed the best suitability for fitting the drying data of murtilla berries [68] and kiwi berries [69].

#### 3.2.4. Jambolan Aspects after Drying

In terms of fruit aspects, at the final of the drying process, a great number of fissures and cracks occurred at the surface level among PT and NPT treatments. Similarly, significant changes have been reported in cranberries subjected to microwave vacuum pretreatment (100–800 W), followed by OD and microwave vacuum drying at a microwave power of 100 W [44]. Liu et al. [70] also observed these changes on the surface of blueberries during drying, using innovative far-infrared radiation heating-assisted pulsed vacuum drying.

### 3.3. Monitoring Analyses of Jambolan after Drying

Table 5 shows the results for moisture content (MC), water activity (a_w_), total monomeric anthocyanins (TMA), and total phenolic contents (TPC) performed in the jambolan after the drying process at different temperatures for the NPT and PT jambolan.

#### 3.3.1. Effect of Osmotic Pretreatment and Drying on Moisture Contents and Water Activities

The PVOD combined with the air-drying showed that both processes are useful tools to reduce even further the MC and a_w_ of jambolan, in comparison to dried jambolan with no pretreatment, at each temperature studied. In general, an increase in temperature promoted a decrease in the MC and a_w_ values for NPT and PT jambolan. Regarding MC, no statistical difference was noted between NPT (50 °C) and NPT (60 °C), nor between PT (60 °C) and PT (70 °C) (*p* > 0.05). For a_w_, all treatments were statistically different from each other (*p* ≤ 0.05). The fruit’s initial MC and a_w_ are comparable to the results found in jambolan from the Amazonian region [26,42]. For dried papaya slices, Chandra et al. [71] found MC and a_w_ variations around 7.6–12.5% and 0.39–0.45, respectively, using ultrasonic and osmotic pretreatments, followed by convective and vacuum drying.

There are several reasons for food products to undergo OD and drying techniques—one of them is the need to preserve them by lowering the a_w_, since microorganisms grow, sporulate, and germinate at different a_w_ levels. Pretreatment of foods before drying stages may be responsible for inactivating microorganisms and enzymes. Removal of water by drying reduces the a_w_ as it tends to exhibit a negative effect on the growth and survival of different microorganisms, retarding the deterioration in foods, which represents a critical step in producing safe food products. Depending on the temperature and drying exposure time, a certain number of microbial cells can die or obtain sub-lethal injuries [41,72].

The high a_w_ values observed in NPT and PT jambolan fruits (Table 5) are commonly noticed in high-perishable foods. At this region (a_w_ > 0.95), microorganisms such as *Pseudomonas, Escherichia, Proteus, Shigella, Klebisiella, Bacillus, Clostridium perfringens*, and some yeasts are frequently found in fruit and vegetables [73]. For NPT jambolan dried at 60 °C, most of the halophilic bacteria and mycotoxigenic *Aspergillus* can be found (0.75 < a_w_ < 0.8). Additionally, xerophilic fungi (*Aspergillus chevalieri, candidus, Wallemia Sebi*) and *Saccharomyces bisporus* can represent a potential growth in NPT and PT jambolan and dried at 50 °C (a_w_ between 0.65 and 0.75) [73]. Microbial growth does not occur at a_w_ < 0.60 [74]. Therefore, only NPT jambolan dried at 70 °C and PT jambolan dried at 60 and 70 °C reached values lower than the a_w_ limit for establishing microbiological food safety.

#### 3.3.2. Effect of Osmotic Pretreatment and Drying on Total Monomeric Anthocyanins and Total Phenolic Contents

NPT samples suffered decreases in the TMA and TPC throughout the air-drying, at different temperature conditions. In general, these bioactive compound losses were continually increasing as the temperature increased (Table 5). For NPT dried jambolan, TMA and TPC retentions were 17% and 99.5% at 50 °C, 16.8% and 77.1% at 60 °C, and 14.3% and 84.2% at 70 °C, respectively. Other studies demonstrated that drying at high temperatures promotes a reduction in the TMA and TPC levels, as observed in strawberries [75], in which 26% TMA and 60.9% TPC had been lost at 50 °C, while at 60 °C, the losses reached 45% and 78.1%, respectively. Liu et al. [70] reported this same phenomenon in blueberries during far-infrared radiation heating-assisted pulsed vacuum drying. These researchers noticed that the TMA and TPC presented a continuous decline with the constant rise of the drying temperature used (65, 70, and 75 °C).

Temperature is one of the main variables affecting anthocyanins’ stability. Degradation by heat is not only dependent on temperature but also depends on processing time, which is mainly caused by oxidation and cleavage of the covalent bonds. The flavylium cation can be transformed into a quinoidal base and, later, into several intermediates, resulting in coumarin derivatives and compounds equivalent to the B ring. Alternatively, flavylium cation could be transformed into a colorless carbinol base and further into chalcone, which results in brown degradation products. Another possible explanation of anthocyanin degradation occurs similarly to the last-mentioned mechanism; however, the molecule deglycolysis occurs first, leading to the loss of glycosidic bond and the formation of unstable anthocyanins, whose degradation products express brown and yellow colors [76].

Furthermore, at higher temperatures, a reduction in the TPC during drying can be described by the release of bound phenolic compounds and partial degradation of lignin, which promotes the release of phenolic acid derivatives [77]. Parallel to the chemical changes, physical alterations, such as collapse, shrinkage, and porosity, can favor the exposure of the compounds to oxygen and light. Therefore, the overall effect of thermal processing on bioactive compounds retention is dependent on the complexity of physical and chemical phenomena [75].

Concerning the PT dried jambolan, the lowest retention of bioactive compounds was found at 50 °C, which corresponded to 9.1% TMA and 61.5% TPC. These results show that although drying has been carried out at a low temperature, the prolonged drying time promoted the strongest degradation. The highest TMA and TPC retentions were recorded at 60 °C, which were equivalent to 18.0% and 116.5%, respectively. Garba et al. [53] also recorded the highest MA values for black carrots dried at 60 °C in both control and blanched samples (98 °C for 3 min). Still, as shown in Table 5, when the temperature increased from 60 °C to 70 °C, the retention of the bioactive compounds decreased again, accounting for 15.7% TMA and 94.2% TPC, with regards to the initial PT contents. Therefore, in this process condition, the drying temperature was predominant for the degradation of TMA and TPC when compared to the drying time.

Tan et al. [78] state that drying temperature and time can affect the quality of dried fruits and vegetables. When the drying temperature is too low, enzymatic browning increases, and if the temperature is too high, most of the nutrients can be destroyed. In PT jambolan, it is possible that the ML phenomenon promoted a change in cell membrane integrity and resulted in a loss of compartmentation of enzymes and substrates, permitting their interaction. Nunes et al. [79] have attributed the TMA and TPC losses to the accelerated ML during the storage of strawberries, in which the oxidation of phenolic compounds, by polyphenol oxidase (PPO), might have induced the formation of *o*-quinones and stimulated the anthocyanin degradation by coupled oxidation mechanisms. According to Maghsoudlou et al. [80], phenolic compounds (as chlorogenic acid) are one the main substrates of the catecholase activity from PPO, and the oxidation process can alter the concentration of these phenolic compounds.

From the perspective of presenting a shelf-stable product during storage, PT jambolan dried at 60 °C appears as a good option, since the a_w_ level lies within the permitted limit for microbial safety. Therefore, the osmotic pretreatment, although it decreased the bioactive compound contents, it represented a promising path to preserve these nutrients, since TMA and TPC values remained very similar at the final drying stage. Still, this process condition demands less drying time in comparison with the NPT dried products (Figure 4), and thus it favors the reduction of drying costs because of energy savings.

In the end, the jambolan fruit has a formidable potential for the food and pharmaceutical industry sectors. On the other hand, the lack of investments in technologies for its post-harvesting increases the rate of residues, resulting in a non-stimulating scenario for the cultivation and commercialization of the fruit [7]. The present work, however, provides a promising contribution to avoiding the fruit’s waste during the seasonal period, making it available for much longer by presenting a quality product under the levels required for avoiding microbial growth.

## 4. Conclusions

Combination of pulsed vacuum osmotic dehydration (PVOD) and hot air-drying process was studied for the first time in jambolan fruits. PVOD resulted in partial water removal from the fruit, with little solid uptake. Nevertheless, PVOD decreased the anthocyanins and total phenolic contents throughout time, which could be induced by leaching into the osmotic solution or either by chemical or biochemical reactions. The rise in air-drying temperature shortened the drying time for both non-pretreated and osmotically pretreated jambolan. In addition, osmotic pretreatment lowered the energy demands for posterior drying of jambolan, which is interesting in terms of economic issues. Furthermore, the fruit pretreated by PVOD and dried at 60 °C showed promising results, due to its low moisture content and a_w_, as well as its TMA and TPC contents, which were nearly the same as in the non-pretreated sample (control), dried at 60 °C. Finally, this study presents practical data for industrial purposes, as well as a good alternative to prolong the shelf life of the jambolan fruit and make it available for much longer.

## Figures and Tables

**Table 1:** Models tested for air-drying conditions of NPT and PT jambolan.

Model	Mathematical Equation	Equation
Newton	MR=exp(-kt)	(14)
Page	$\mathrm{MR}=\exp\left({-\mathrm{kt}}^{n}\right)$	(15)
Modified Page	$\mathrm{MR}=\exp[-{\left(\mathrm{kt}\right)}^{n}]$	(16)
Henderson and Pabis	MR=a exp − kt	(17)
Logarithm	MR=a exp(-kt)+c	(18)
Midilli	$\mathrm{MR}=a\;\exp\left({-\mathrm{kt}}^{n}\right)+\mathrm{bt}$	(19)
Two-term	$\mathrm{MR}=a\;\exp\left({-k}_{0}t\right)+b\;\exp\left({-k}_{1}t\right)$	(20)
Diffusion approximation	MR=a exp(-kt)+(1 - a)exp(-kbt)	(21)
Two-term exponential	MR=a exp(-kt)+(1 - a)exp(-kat)	(22)

MR = moisture ratio (dimensionless); t = drying time (min); k, k_0_, and k_1_ are drying coefficients; a, b, and n are equation fit parameters.

**Table 2:** Parameter values estimated for the models proposed by Azuara and Peleg, fitted to the PVOD kinetic data for jambolan.

Moisture Loss					Solid Gain				
Azuara’s Model									
k		ML^ꝏ^	R^2^	RMSE	k		SG^ꝏ^	R^2^	RMSE
0.09		61.73	1	0.66	0.17		5.95	0.98	0.23
Peleg’s Model									
k_1_	k_2_	ML^ꝏ^	R^2^	RMSE	k_1_	k_2_	SG^ꝏ^	R^2^	RMSE
0.19	0.02	61.99	0.99	0.53	0.73	0.18	5.46	0.58	0.22

ML^∞^ = moisture loss at equilibrium (%); SG^∞^ = solid gain at equilibrium (%); k = Azuara’s constant (h^−1^); k_1_ and k_2_ = Peleg’s constants; R^2^ = coefficient of determination; RMSE = root mean square error.

**Table 3:** Effective moisture diffusivities (D_eff_) and activation energy (E_a_) for the drying processes of non-pretreated and osmotically pretreated jambolan fruits.

Temperature (°C)	D_eff_ (m^2^/s)	E_a_ (kJ/mol)
Non-pretreated		
50	1.45 × 10^−10^	52.0
60	3.85 × 10^−10^	
70	4.46 × 10^−10^	
Pretreated		
50	2.56 × 10^−10^	30.2
60	3.99 × 10^−10^	
70	4.93 × 10^−10^	

**Table 4:** Values of the coefficient of determination (R^2^), reduced chi-square (χ^2^), root mean square error (RMSE), and residue dispersion (RD) for the non-linear regression of the models fitted to the drying kinetic data of jambolan fruits.

Model	Statistics											
	50 °C				60 °C				70 °C			
	R^2^	χ^2^ (×10^−4^)	RMSE (×10^−2^)	RD	R^2^	χ^2^ (×10^−5^)	RMSE (×10^−2^)	RD	R^2^	χ^2^ (×10^−5^)	RMSE (×10^−2^)	RD
Non-pretreated												
1	0.97	25	5	B	0.99	13	3	B	0.99	15	4	B
2	0.99	14	4	B	0.99	10	3	B	1	3	4	R
3	0.99	14	4	B	0.99	9	3	B	1	3	2	R
4	0.98	24	5	B	0.99	13	3	B	0.99	12	3	B
5	1	1	1	B	1	4	2	R	1	3	1	B
6	1	0.4	1	R	1	2	1	R	1	1	1	R
7	0.98	25	5	B	0.99	14	3	B	0.99	14	3	B
8	1	3	2	B	1	3	2	R	1	2	1	B
9	0.99	14	4	B	0.99	10	3	B	1	3	2	R
Pretreated												
1	0.98	21	5	B	0.99	16	4	B	0.99	11	3	R
2	0.99	7	3	B	0.99	9	3	B	1	5	2	R
3	0.99	7	3	B	0.99	9	3	B	1	5	2	R
4	0.98	18	4	B	0.99	16	4	B	0.99	9	3	R
5	1	0.3	1	R	1	1	1	R	1	2	1	R
6	1	0.2	0.4	R	1	1	1	R	1	2	1	R
7	0.99	7	2	B	0.99	18	4	B	1	2	1	R
8	1	0.2	0.4	R	1	2	1	B	1	5	2	R
9	0.99	8	3	B	0.99	8	3	B	1	5	2	R

Model: (1) Newton; (2) Page; (3) Modified Page; (4) Henderson and Pabis; (5) Logarithm; (6) Midilli; (7) two-term; (8) diffusion approximation; (9) two-term exponential. RD: residues dispersion; R: random; B: biased.

**Table 5:** Moisture content (MC), water activity (a_w_), total monomeric anthocyanins (TMAs), and total phenolic contents (TPCs) at the final drying time for the non-pretreated and osmotically pretreated jambolan fruits.

Jambolan Treatments	MC	a_w_	TMA	TPC
NPT	87.32 ± 0.13 ^a^	0.980 ± <0.001 ^a^	477.89 ± 16.83 ^a^	1236.13 ± 57.56 ^a^
NPT and dried at 50 °C	29.78 ± 0.26 ^c^	0.712 ± 0.001 ^d^	81.24 ± 5.33 ^c^	1230.30 ± 34.42 ^ab^
NPT and dried at 60 °C	28.34 ± 0.77 ^c^	0.758 ± 0.001 ^c^	80.02 ± 0.10 ^c^	952.86 ± 43.38 ^c^
NPT and dried at 70 °C	20.88 ± 0.26 ^e^	0.582 ± 0.001 ^g^	68.31 ± 7.97 ^cd^	1041.30 ± 5.67 ^bc^
PT	69.38 ± 0.56 ^b^	0.959 ± 0.001 ^b^	255.35 ± 15.90 ^b^	906.80 ± 101.09 ^c^
PT and dried at 50 °C	24.21 ± 0.46 ^d^	0.672 ±< 0.001 ^e^	23.21 ± 2.38 ^d^	557.88 ± 36.92 ^d^
PT and dried at 60 °C	17.34 ± 0.33 ^f^	0.599 ± 0.001 ^f^	46.06 ± 1.41 ^cd^	1056.22 ± 15.19 ^abc^
PT and dried at 70 °C	17.79 ± 0.76 ^f^	0.555 ± 0.001 ^h^	40.08 ± 1.57 ^cd^	854.55 ± 20.78 ^c^

MC: moisture content (g/100 g wet basis—wb); a_w_: water activity (dimensionless); TMA: total monomeric anthocyanin (mg delphinidin-3-glucoside/100 g fruit db); TPC: total phenolic content (mg GAE/100 g fruit db); NPT: non-pretreated; PT: pretreated. Values in the same column with different superscript letters are significantly different by Tukey’s test (*p* ≤ 0.05).

## Data Availability

Data are contained within the article.

## References

[1] Singh J.P., Kaur A., Singh N., Nim L., Shevkani K., Kaur H., Arora D.S. (2016). In vitro antioxidant and antimicrobial properties of jambolan (*Syzygium cumini*) fruit polyphenols. LWT-Food Sci. Technol..

[2] Patras A., Brunton N., O’Donnel C., Tiwari B.K. (2010). Effect of thermal processing on anthocyanin stability in foods; mechanisms and kinetics of degradation. Trends Food Sci. Technol..

[3] Limsitthichaikoon S., Saodaeng K., Priprem A., Damrongrunggrang T. (2015). Anthocyanin complex: Characterization and cytotoxicity studies. Int. J. Chem. Mol. Eng..

[4] Lestario L.N., Howard L.R., Brownmiller C., Stebbins N.B., Liyanage R., Lay J.O. (2017). Changes in polyphenolics during maturation of Java plum (*Syzygium cumini* Lam.). Food Res. Int..

[5] Singh B., Singh J.P., Kaur A., Singh N. (2018). Insights into the phenolic compounds present in jambolan (*Syzygium cumini*) along with their health-promoting effects. Int. J. Food Sci. Technol..

[6] Silva W.P., Nunes J.S., Gomes J.P., Araújo A.C., Silva C.M.D.P.S. (2018). Description of jambolan (*Syzygium cumini* (L.)) anthocyanin extraction kinetics at different stirring frequencies of the medium using diffusion models. Heat Mass Transf..

[7] Sabino L.B.S., Brito E.S., Silva-Júnior I.J., Rodrigues S., Silva E.O., Brito E.S. (2018). Jambolan—*Syzygium Jambolanun*. Exotic Fruits.

[8] Hssaini L., Ouaabou R., Charafi J., Idlimam A., Lamharrar A., Razouk R., Hanine H. (2022). Hygroscopic proprieties of fig (*Ficus carica* L.): Mathematical modelling of moisture sorption isotherms and isosteric heat kinetics. S. Afr. J. Bot..

[9] Karam M.C., Petit J., Zimmer D., Baudelaire Djantou E., Scher J. (2016). Effects of drying and grinding in production of fruit and vegetable powders. J. Food Eng..

[10] Pandiselvam R., Tak Y., Olum E., Sujayasree O.J., Tekgül Y., Koç G.Ç., Kaur M., Nayi P., Kothakota A., Kumar M. (2022). Advanced osmotic dehydration techniques combined with emerging drying methods for sustainable food production: Impact on bioactive components, texture, color, and sensory properties of food. J. Texture Stud..

[11] Manzoor A., Khan M.A., Mujeebu M.A., Shiekh R.A. (2021). Comparative study of microwave assisted and conventional osmotic dehydration of apple cubes at constant temperature. J. Agri. Food Res..

[12] Yadav A.K., Singh S.V. (2014). Osmotic dehydration of fruits and vegetables: A review. J. Food Sci. Technol..

[13] Ramya V., Jain N.K. (2016). A review on osmotic dehydration of fruits and vegetables: An integrated approach: Osmo-dehydration of fruits and vegetables. J. Food Process Eng..

[14] Ma Y., Yi J., Bi J., Zhao Y., Li X., Wu X., Du Q. (2021). Effect of ultrasound on mass transfer kinetics and phenolic compounds of apple cubes during osmotic dehydration. LWT-Food Sci. Technol..

[15] Carmo J.R., Corrêa J.L.G., Resende M., Cirillo M.A., Corona-Jiménez E., Telis-Romero J. (2022). Mango enriched with sucrose and isomaltulose (Palatinose^®^) by osmotic dehydration: Effect of temperature and solute concentration through the application of multilevel statistical models. J. Food Process. Preserv..

[16] Macedo L.L., Corrêa J.L.G., Petri Júnior I., Araújo C.S., Vimercati W.C. (2022). Intermittent microwave drying and heated air drying of fresh and isomaltulose (Palatinose) impregnated strawberry. LWT-Food Sci. Technol..

[17] Macedo L.L., Corrêa J.L.G., Vimercati W.C., Araújo C.S. (2022). The impact of using vacuum and isomaltulose as an osmotic agent on mass exchange during osmotic dehydration and their effects on qualitative parameters of strawberries. J. Food Process Eng..

[18] Silva K.S., Fernandes M.A., Mauro M.A. (2017). Effect of calcium on the osmotic dehydration kinetics and quality of pineapple. J. Food Eng..

[19] Tappi S., Mauro M.A., Tylewics U., Dellarosa N., Della Rosa M., Rocculi P. (2017). Effects of calcium lactate and ascorbic acid on osmotic dehydration kinetics and metabolic profile of apples. Food Bioprod. Process..

[20] Sahin U., Öztürk H.K. (2016). Effects of pulsed vacuum osmotic dehydration (PVOD) on drying kinetics of figs (*Ficus carica* L). Innov. Food Sci. Emerg. Technol..

[21] Junqueira J.R.J., Corrêa J., Mendonça K., Mello R.E. (2018). Pulsed vacuum osmotic dehydration of beetroot, carrot and eggplant slices: Effect of vacuum pressure on the quality parameters. Food Bioproc. Technol..

[22] Mello R., Corrêa J.L.G., Lopes F.J., Souza A.U., Silva K.C.R. (2019). R. Kinetics of the pulsed vacuum osmotic dehydration of green fig (*Ficus carica L*.). Heat Mass Transf..

[23] Junqueira J.R.J., Corrêa J.L.G., Mendonça K.S., Mello Júnior R.E., Souza A.U. (2021). Modeling mass transfer during osmotic dehydration of different vegetable structures under vacuum conditions. Food Sci. Technol..

[24] Ghellam M., Zannou O., Galanakis C.M., Aldawoud T.M.S., Ibrahim S.A., Koca I. (2021). Vacuum-assisted osmotic dehydration of autumn olive berries: Modeling of mass transfer kinetics and quality assessment. Foods.

[25] Sharma M., Dash K.K. (2019). Effect of ultrasonic vacuum pretreatment on mass transfer kinetics during osmotic dehydration of black jamun fruit. Ultrason. Sonochem..

[26] Araújo A.L., Pena R.S. (2022). Influence of process conditions on the mass transfer of osmotically dehydrated jambolan fruits. Food Sci. Technol..

[27] Kucner A., Klewicki R., Sójka M. (2013). The influence of selected osmotic dehydration and pretreatment parameters on dry matter and polyphenol content in highbush blueberry (*vaccinium corymbosum* l.) fruits. Food Bioproc. Technol..

[28] Sridevi M., Genitha E.T.R. (2012). Optimization of osmotic dehydration process of pineapple by response surface methodology. J. Food Process. Technol..

[29] AOAC (2002). Official Methods of Analysis of AOAC International.

[30] Brito B.N.C., Pena R.S., Lopes A.S., Chisté R.C. (2017). Anthocyanins of jambolão (*Syzygium cumini*): Extraction and pH-dependent color changes. J. Food Sci..

[31] Singleton V.L., Rossi J.A. (1965). Colorimetry of total phenolic with phosphomolybdic-phosphotungstic acid reagents. Am. J. Enol. Vitic..

[32] Giusti M.M., Wrolstad R.E. (2001). Characterization and measurement with UV-visible spectroscopy. Curr. Protoc. Food Anal. Chem..

[33] Azuara E., Cortés R., Garcia H.S., Beristain C.I. (1992). Kinetic model for osmotic dehydration and its relationship with Fick’s second law. Int. J. Food Sci. Technol..

[34] Peleg M. (1988). An empirical model for the description of moisture curves. J. Food Sci..

[35] Crank J. (1975). The Mathematics of Diffusion.

[36] Xanthopoulos G., Yanniotis S., Lambrinos G. (2009). Water diffusivity and drying kinetics of air drying of figs. Dry. Technol..

[37] Sacilik K., Elicin A.K., Unal G. (2006). Drying kinetics of Üryani plum in a convective hot air-drying. J. Food Eng..

[38] Akpinar E.K., Bicer Y., Yildiz C. (2003). Thin layer drying of red pepper. J. Food Eng..

[39] Rastogi N.K., Raghavarao K.S.M.S. (1997). Water and solute diffusion coefficients of carrot as a function of temperature and concentration. J. Food Eng..

[40] Rastogi N.K., Raghavarao K.S.M.S., Niranjan K., Knorr D. (2002). Recent development in osmotic dehydration: Methods to enhance mass transfer. Trends Food Sci. Technol..

[41] Erkmen O., Bozoglu T.F., Erkmen O., Bozolgu T.F. (2016). Food preservation by reducing water activity. Food Microbiology: Principles into Practice.

[42] Araújo A.L., Pena R.S. (2022). Moisture desorption behavior and thermodynamic properties of pulp and seed of jambolan (*Syzygium cumini*). Heliyon.

[43] Junqueira J.R.J., Corréa J.G., Mendonça K.S. (2016). Evaluation of the shrinkage effect on the modeling kinetics of osmotic dehydration of sweet potato (*Ipomoea batatas* (L.)). J. Food Process. Preserv..

[44] Zielinska M., Zielinska D., Markowski M. (2018). The effect of microwave-vacuum pretreatment on the drying kinetics, color and the content of bioactive compounds in osmo-microwave-vacuum dried cranberries (*Vaccinium macrocarpon*). Food Bioproc. Technol..

[45] Yu Y., Jin T.Z., Fan X., Wu J. (2018). Biochemical degradation and physical migration of polyphenolic compounds in osmotic dehydrated blueberries with pulsed electric field and thermal pretreatment. Food Chem..

[46] Evert R.F., Eichhorn S.E. (2013). Raven Biology of Plants.

[47] Nowicka P., Wojdyło A., Lech K., Figiel A. (2015). Influence of osmodehydration pretreatment and combined drying method on the bioactive potential of sour cherry fruits. Food Bioproc. Technol..

[48] Araya-Farias M., Macaigme O., Ratti C. (2014). On the development of osmotically dehydrated seabuckthorn fruits: Pretreatments, osmotic dehydration, post drying techniques, and nutritional quality. Dry. Technol..

[49] Sójka A., Karlinska E., Klewicki R. (2017). Ellagitannin and anthocyanin retention in osmotically dehydrated blackberries. Food Sci. Technol. Res..

[50] Blanda G., Cerretani L., Cardinali A., Barbieri S., Bendini A., Lercker G. (2009). Osmotic dehydrofreezing of strawberries: Polyphenolic content, volatile profile and consumer acceptance. LWT-Food Sci. Technol..

[51] Souraki B.A., Ghavami M., Tondro H. (2014). Simulation of osmotic dehydration of a spherical material using parabolic and powerlaw approximation methods. Chem. Eng. Commun..

[52] Krokida M.K., Maroulis Z.B. (2001). Structural properties of dehydrated products during rehydration. Int. J. Food Sci. Technol..

[53] Garba U., Kaur S., Gurumayum S., Rasane P. (2015). Effect of hot water blanching time and drying temperature on the thin layer drying kinetics of and anthocyanin degradation in black carrot (*Daucus carota* L.) shreds. Food Technol. Biotechnol..

[54] Potosí-Calvache D.C., Vanegas-Mahecha P., Martínez-Correa H.A. (2017). Convective drying of squash (*Cucurbita moschata*): Influence of temperature and air velocity on effective moisture diffusivity, carotenoid content and total phenols. DYNA.

[55] An K., Li H., Zhao D., Ding S., Tao H., Wang Z. (2013). Effect of osmotic dehydration with pulsed vacuum on hot-air drying kinetics and quality attributes of cherry tomatoes. Dry. Technol..

[56] Fito P., Chiralt A., Barat J.M., Andrés A., Martínez-Monzo J., Martinéz-Navarrete N. (2001). Vacuum impregnation for development of new dehydrated products. J. Food Eng..

[57] Madamba P.S., Griscoll R.H., Buckle K.A. (1996). The thin layer drying characteristics of garlic slices. J. Food Eng..

[58] Barathiraja R., Thirumal P., Thirumalaikumarasamy D., Kajavali A., Ashokkumar M., ThiyagaRaj J. (2021). Investigation of drying kinetics and qualities of turkey berry in fluidized bed dryer. Mater. Today Proc..

[59] Martín-Gómez J., Ángeles Varo M., Mérida J., Serratosa M.P. (2020). Influence of drying processes on anthocyanin profiles, total phenolic compounds and antioxidant activities of blueberry (*Vaccinium corymbosum*). LWT-Food Sci. Technol..

[60] Falade K.O., Oyedele O.O. (2010). Effect of osmotic pretreatment on air drying characteristics and colour of pepper (*Capsicum* spp) cultivars. J. Food Sci. Technol..

[61] Xiao H.W., Pang C.L., Wang L.H., Bai J.W., Yang W.X., Gao Z.J. (2010). Drying kinetics and quality of Monukka seedless grapes dried in an air-impingement jet dryer. Biosyst. Eng..

[62] Amami E., Khezami W., MezriguI S., Badwaik L.B., Bejar A.K., Perez C.T., Kechaou N. (2017). Effect of ultrasound-assisted osmotic dehydration pretreatment on the convective drying of strawberry. Ultrason. Sonochem..

[63] Purlis E. (2019). Modelling convective drying of foods: A multiphase porous media model considering heat of sorption. J. Food Eng..

[64] Babalis S.J., Belessiotis V.G. (2004). Influence of the drying conditions on the drying constants and moisture diffusivity during the thin-layer drying of figs. J. Food Eng..

[65] Wiktor A., Iwaniuk M., Śledź M., Nowacka M., Chudoba T., Witrowa-Rajchert D. (2013). Drying kinetics of apple tissue treated by pulsed electric field. Dry. Technol..

[66] Wisniak J., Polishuk A. (1999). Analysis of residuals—A useful tool for phase equilibrium data analysis. Fluid Ph. Equilibria.

[67] Assis F.R., Morais R.M.S.C., Morais A.M.M.B. (2017). Mathematical modelling of osmotic dehydration kinetics of apple cubes. J. Food Process. Preserv..

[68] Puente-Díaz L., Ah-Hen K., Veja-Gálvez A., Lemus-Mondaca R., Scala K.D. (2013). Combined infrared-convective drying of murta (*Ugni molinae* Turcz) berries: Kinetic modeling and quality assessment. Dry. Technol..

[69] Bialik M., Wiktor A., Witrowa-Rajchert D., Gondek E. (2020). The influence of osmotic dehydration conditions on drying kinetics and total carotenoid content of kiwiberry (*actinidia arguta*). Int. J. Food Eng..

[70] Liu Z.L., Xie L., Zielinska M., Pan Z., Deng L., Zhang J., Gao L., Wang S., Zheng Z., Xiao H. (2022). Improvement of drying efficiency and quality attributes of blueberries using innovative far-infrared radiation heating assisted pulsed vacuum drying (FIR-PVD). Innov. Food Sci. Emerg. Technol..

[71] Chandra A., Kumar S., Tarafdar A., Nema P.K. (2020). Ultrasonic and osmotic pretreatments followed by convective and vacuum drying of papaya slices. J. Sci. Food Agric..

[72] Kowalska H., Marzec A., Kowalska J., Trych U., Masiarz E., Lenart A. (2020). The use of a hybrid drying method with pre-osmotic treatment in strawberry bio-snack technology. Int. J. Food Eng..

[73] Reid D.S., Fennema O., Damodaran S., Parkin J.L., Fennema O. (2008). Water and ice. Fennema’s Food Chemestry.

[74] Scott W.J. (1957). Water relations of food spoilage microorganisms. Adv. Food Res..

[75] Méndez-Lagunas L., Rodríguez-Ramírez J., Cruz-Gracida M., Sandoval-Torres S., Barriada-Bernal G. (2017). Convective drying kinetics of strawberry (*Fragaria ananassa*): Effects on antioxidant activity, anthocyanins and total phenolic content. Food Chem..

[76] Schwartz S.J., Cooperstone J.L., Cichon M.J., Von Elbe J.H., Giusti M., Damodaran S., Parkin K.L. (2017). Colorants. Fennema’s Food Chemistry.

[77] Maillard M.N., Berset C. (1995). Evolution of antioxidant activity during kilning: Role of insoluble bound phenolic acids of barley and malt. J. Agric. Food Chem..

[78] Tan S., Miao Y., Zhou C., Luo Y., Lin Z., Xie R., Li W. (2022). Effects of hot air drying on drying kinetics and anthocyanin degradation of blood-flesh peach. Foods.

[79] Nunes M.C.N., Brecht J.K., Morais A.M.B., Sargent S.A. (2005). Possible influences of water loss and polyphenol oxidase activity on anthocyanin content and discoloration in fresh ripe strawberry (CV. *Oso grande*) during storage at 1 °C. J. Food Sci..

[80] Maghsoudlou Y., Guajari M.A., Tavasoli S. (2019). Effects of heat treatment on the phenolic compounds and antioxidant activity of quince fruit and its tisane’s sensory properties. J. Food Sci. Technol..

